# Outcome and Quality of Life in Guillain–Barré Syndrome in Southern Brazil

**DOI:** 10.1155/nri/1018409

**Published:** 2026-10-22

**Authors:** Giuliana Pipolo, Lara Santos Godoy, Eduardo Hummelgen, Carlos Arteaga Rodriguez, Otto Jesus Hernandez Fustes

**Affiliations:** ^1^ Neurology Service Hospital do Rocio Campo Largo Brazil; ^2^ Federal University of Paraná Curitiba Brazil ufpr.br; ^3^ Positive University and Pontifical Catholic University of Paraná Curitiba Brazil; ^4^ Clinical Hospital Complex at Federal University of Paraná Curitiba Brazil

**Keywords:** acute polyradiculoneuritis, Guillain–Barré syndrome, neuromuscular disorders, quality of life

## Abstract

**Background:**

Guillain–Barré Syndrome (GBS) is an acute immune‐mediated polyradiculoneuropathy with an annual incidence of 0.4 per 100,000 inhabitants in Brazil. Clinical manifestations range from mild weakness to tetraplegia with respiratory failure.

**Objective:**

To analyze the impact on the quality of life of adult patients diagnosed with GBS in the short and medium term.

**Methods:**

This regional descriptive, cross‐sectional study included 46 hospitalized patients with confirmed GBS. Functional status was measured using the overall disability sum score (ODSS), and quality of life was evaluated with the WHOQOL‐BREF instrument.

**Results:**

During the study period, 57 patients were diagnosed with GBS; 11 met exclusion criteria, and 5 died, yielding a mortality rate of 8.7%. All deceased patients were older than 55 years (mean 59,6 *p* = 00, 008) and presented with respiratory failure. Higher Hughes scores at admission (mean 3.8 vs. 2.75; *p* = 0.039) and longer duration of antecedent symptoms (24.33 vs. 14.80 days; *p* = 0.036)were significantly associated with mortality. Moderate/high‐risk mEGOS scores were present in 80% of deceased patients (*p* = 0.008). Quality of life perceptions improved most between 6 and 12 months postdiagnosis, while functional recovery (ODSS) was observed after 12 months.

**Conclusion:**

Higher admission neurological disability was associated with greater functional impairment in this cohort, while quality of life improved during follow‐up and functional recovery continued beyond 12 months. Higher Hughes scores and moderate‐to‐high mEGOS risk were associated with mortality in univariable analyses, but the small number of deaths and absence of multivariable adjustment preclude conclusions regarding independent prognostic effects. Extended follow‐up is important to capture the gradual trajectory of functional and psychosocial recovery in GBS.

## 1. Introduction

Guillain–Barré Syndrome (GBS) is an acute immune‐mediated polyradiculoneuritis, preceded by an infectious condition in 70% of cases [1].

Epidemiological data have shown an incidence of approximately 0.4 cases per 100,000 inhabitants per year in Brazil, with the highest frequency observed during the summer months, when the combination of elevated temperatures and increased rainfall predisposes the population to infectious conditions that can potentially initiate GBS [2].

The clinical presentation of GBS is remarkably heterogeneous. Some patients present with mild distal lower limb (LL) paraparesis that does not prevent them from walking and recover spontaneously after 4 weeks; however, there are cases that develop severe tetraplegia, with dysautonomia symptoms and respiratory failure, which can lead to death or long‐term sequels [3].

GBS has an efficient and well‐established treatment and generally has a good prognosis; however, 5% of patients die, and around 20% remain with motor sequelae and inability to walk independently 6 months to 1 year after the event [4].

The residual impairments not only affect physical function but also have profound implications for the psychological and emotional well‐being of survivors, who may experience depression and anxiety as a consequence of the acute illness and its aftermath.

Therefore, it is crucial to evaluate in detail the short‐, medium‐, and long‐term prognosis of these patients in our population, in order to identify factors that lead to better and worse prognosis, and, in addition, to evaluate the quality of life (QoL) of these patients, considering the degree of physical impairment and the emotional damage involved.

Understanding the comprehensive outcomes and QoL of GBS patients is crucial for developing more effective therapeutic strategies, improving rehabilitation protocols, and addressing the holistic needs of individuals affected by this condition. Studies focusing on functional recovery must consider both physical and psychological domains, as the impact of GBS extends beyond the motor symptoms to affect social and emotional functioning. This dual focus is essential for tailoring patient management and postdischarge care, with the ultimate goal of improving both the functional and psychological recovery of GBS patients.

## 2. Methods

We conducted a regional descriptive, cross‐sectional outcome study based on retrospective review of medical records, complemented by follow‐up telephone interviews. The study participants comprised adult patients admitted to the neurology ward at Hospital do Rocio, located in southern Brazil, with an initial diagnostic hypothesis of acute flaccid paralysis or polyradiculoneuritis and without evidence of other neurological deficits, pre‐existing neurological conditions, or motor disabilities.

### 2.1. Study Population and Case Ascertainment

Between January 1, 2022, and April 30, 2024, seventy‐six patients aged ≥ 18 years were admitted to Hospital do Rocio with a provisional diagnosis of GBS established by emergency department physicians.

### 2.2. Diagnostic Confirmation

All patients underwent subsequent evaluation by the neurology service. A confirmed diagnosis of GBS was established in 57 cases, based on the criteria proposed by Asbury and Cornblath (1990) [5] and the Brighton Criteria [6].

### 2.3. Exclusion Criteria

Of the 57 confirmed cases, 11 patients were excluded due to severe pre‐existing motor or sensory disorders, which would have compromised the functional assessments required by the study protocol. Specifically, six patients presented with advanced hip osteoarthritis, chronic pain secondary to traumatic sequelae, or motor deficits following prior cerebrovascular accident, while five had critical illness polyneuropathy associated with diabetes mellitus.

### 2.4. Final Cohort and Follow‐Up

As shown in Figure 1, a total of 46 patients with confirmed GBS were enrolled in the study. Following hospital discharge, five did not return for follow‐up. Thus, of the initial 76 patients with suspected GBS, 19 received alternative diagnoses after specialist evaluation, leaving 57 with confirmed GBS. Of these, 46 were included in the study cohort. At study completion, five patients were lost to follow‐up, and five patients had died.

**FIGURE 1:**
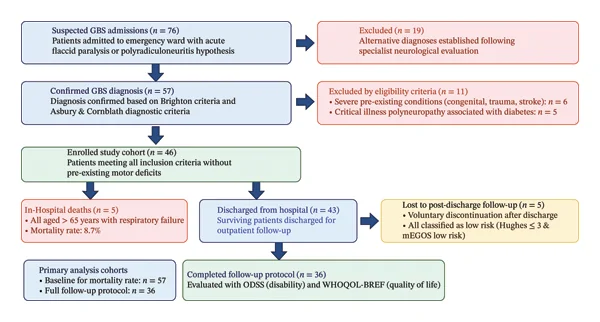
Flowchart illustrating patient selection, diagnostic confirmation, exclusions, and follow‐up trajectory. Of 76 initial patients admitted with suspected Guillain–Barré syndrome (GBS), 19 received alternative diagnoses, and 57 were confirmed using the Brighton and Asbury–Cornblath criteria. Eleven patients were excluded due to severe pre‐existing sensorimotor conditions. Among the 46 enrolled patients, 5 died during hospitalization, and 5 were lost to postdischarge follow‐up. A total of 41 patients were included in baseline comparative and mortality analyses, and 36 completed the full outpatient functional (ODSS) and quality of life (WHOQOL‐BREF) evaluation protocol.

Electroneuromyography was not performed on the patients in this study, as this examination is not widely available in hospitals in the region. We acknowledge that this is a limitation of the study. However, considering countries with economic characteristics similar to those in Brazil, we believe that the study reflects the reality of access to complementary exams that most of the world’s population has. Nevertheless, despite its relationship with prognosis and subtype of GBS, the electromyographic findings do not modify the treatment to be performed nor should alter timing of treatment [7].

The patients were classified according to the modified Erasmus prognostic score–modified Erasmus GBS outcome score (mEGOS)–used to assess the patient’s overall prognosis based on various clinical parameters, such as strength and functional status at the nadir and at follow‐up [8], and also according to the Hughes neurological impairment grading scale, used to classify the severity of neurological impairment at the nadir and at follow‐up, based on the level of functional disability [9].

The variables analyzed were as follows: age at diagnosis, gender, infectious condition that preceded GBS (acute gastroenteritis, dengue, or upper airway infection), symptoms at admission, time between the onset of symptoms and hospital admission, time between the onset of symptoms and start of Immunoglobulin infusion, neurological symptoms at NADIR, length of stay, and associated comorbidities.

All data were computed in an Excel spreadsheet, and during the patients’ follow‐up appointments, two assessment scales related to functionality and QoL were applied. These are the overall disability sum score (ODSS) and the World Health Organization’s abbreviated quality of life questionnaire (WHOQOL‐BREF), which is a validated instrument consisting of 26 items that assess domains of health‐related quality of life (HRQoL). These domains include physical health, psychological well‐being, social relationships, and environment. The WHOQOL‐BREF was developed to provide a cross‐cultural measure of QoL that is applicable across diverse populations and clinical conditions. Its concise format makes it particularly useful in clinical and research settings where time constraints limit the use of longer instruments. Studies have demonstrated its reliability and validity in both general populations and patients with chronic diseases, including neurological disorders such as GBS, where HRQoL assessment is essential to complement objective clinical measures and guide patient‐centered care [10].

### 2.5. Statistical Analysis

These data were used to statistically analyze the impact of GBS on patients’ QoL and identify the main factors associated with worse and better outcomes. The data were analyzed using ANOVA to compare means of quantitative factors (e.g., age, time to symptoms) between outcomes. The chi‐squared test was used to compare qualitative factors such as mEGOS and the presence of comorbidity with mortality rates. Pearson correlation assessed the relationship between ODSS score, Hughes score, and WHOQOL‐BREF score. For temporal comparisons of recovery (< 6 months, 6–12 months, and > 12 months), ANOVA with Tukey’s post hoc test was applied to identify specific differences between groups. Baseline demographic, clinical, and treatment characteristics were analyzed descriptively. Continuous variables were reported as mean ± standard deviation (SD), based on data distribution. Categorical variables were expressed as counts (*n*) and percentages (%). Significance was set at *p* < 0.05.

The study was approved by the Human Research Ethics Committee of the Paraná Workers’ Hospital in Curitiba, Brazil, under CAAE 81604124.2.0000.5225, and the confidentiality of personal and medical data was maintained throughout.

## 3. Results

Among the 46 patients enrolled in this investigation, five died during hospitalization and an additional five were lost to follow‐up after discharge.

It is pertinent to emphasize that the total number of adults diagnosed with GBS at Hospital do Rocio between January 1, 2022, and April 30, 2024, was 57 patients. However, as previously explained, 11 individuals presented with pre‐existing sensorimotor conditions that fell under the exclusion criteria and, for this reason, did not participate in the study. Consequently, the observed mortality rate for GBS in this population was 8.7%.

All five fatalities occurred in patients older than 55 years of age, each of whom exhibited respiratory failure as a clinical manifestation. Of these patients, three had a documented history of cardiovascular disease, while one presented with chronic kidney disease concomitant with Type 2 diabetes mellitus.

In summary, 46 patients were included in the study cohort. At study completion, five patients were lost to follow‐up, and five patients had died.

Among the five patients who were lost to follow‐up after hospital discharge, there was one previously healthy 36‐year‐old woman and four men aged between 28 and 65 years. All of these individuals had been classified as low risk according to both the Hughes prognostic scale and the mEGOS scale, and the patients who discontinued participation did not differ significantly from those who remained in the study with respect to key baseline characteristics (age, sex, disease severity, and comorbidities). Furthermore, the decision to cease follow‐up was voluntary and not directly related to the primary outcome (e.g., mortality or clinical complications).

When analyzing the results of the ODSS score, which measures functional disability, and the quantitative variables, we found that a higher score on the Hughes prognostic scale at admission showed a statistically significant correlation with greater functional impairment (Table 1).

**TABLE 1:** Comparison of quantitative factors according to overall function disability (ODSS).

	ODSS	
	Corr (R)	*p* value
Age	−0.111	0.519
Duration of symptoms that preceded the syndrome	−0.170	0.474
Time from onset of symptoms to hospital admission	−0.170	0.322
Time until the first dose of Ig infusion	−0.083	0.641
Hughes prognostic scale at admission	0.387	**0.020**
WHOQOL‐BREF	−0.553	**< 0.001**

*Note:* Bold values indicate statistical significance.

When analyzing the qualitative variables with the ODSS scale, no statistically relevant correlation was observed.

Considering that the ODSS is divided into two parts—the assessment of the upper limbs and the assessment of the LL—we dissected and evaluated each part independently: The upper limb discriminated ODSS scale (ODSS‐UL) score was normal in 23 patients of the total of 36 patients that completed the study protocol (63.9%), Grade 1 in 5 patients (13.9%), Grade 2 in 1 patient (2.8%), Grade 3 in 1 patient (2.8%), Grade 4 in 5 patients (13.9%), and Grade 5 in 1 patient (2.8%). The ODSS score in LL was normal in 16 patients (44.4%), Grade 1 in 14 patients (38.9%), Grade 3 in 1 patient (2.8%), Grade 4 in 1 patient (2.8%), Grade 6 in 1 patient (2.8%), and Grade 7 in 3 patients (8.3%).

Patients who presented with a score of > 3 on the Hughes prognostic scale upon hospital admission revealed severe impairment on the ODSS‐UL scale (*p* = 0.054).

The results for the lower limb discriminated ODSS scale (ODSS‐LL) are presented in Tables 2 and 3.

**TABLE 2:** Comparison of quantitative factors according to lower limb functional disability (ODSS‐LL).

		Mean	Median	Standard deviation	Min	Max	N	*p* value
WHOQOL‐BREF	Normal	101.9	100	12.1	77	128	16	**0.003**
	Mild	95.1	94.5	16.9	71	122	14	
	Severe	75.0	73	18.8	56	104	6	

Age	Normal	49.8	46	14.3	24	76	16	0.718
	Mild	48.1	43.5	18.0	25	85	14	
	Severe	43.5	44	15.0	26	59	6	

Time until hospital admission (days)	Normal	9.0	8	4.5	3	18	16	**0.048**
	Mild	5.9	4.5	3.9	1	14	14	
	Severe	4.8	4.5	3.0	2	10	6	

Time until IG first dose (days)	Normal	11.3	11.5	4.2	6	19	14	0.181
	Mild	8.6	8	4.4	2	17	14	
	Severe	8.2	7	3.8	5	15	6	

Hughes	Normal	2.4	2	1.0	1	4	16	**0.011**
	Mild	2.6	2	0.8	2	4	14	
	Severe	3.8	4	0.8	3	5	6	

Hospitalization period	Normal	12.9	10	10.7	4	43	16	0.122
	Mild	10.1	10	2.9	6	16	14	
	Severe	29.2	10	44.6	9	120	6	

*Note:* Bold values indicate statistical significance.

**TABLE 3:** ODSS‐LL post hoc *p* values.

		Normal	Mild
WHOQOL‐BREF	Mild	0.451	**0.028**
	Severe	**0.002**	

Time until hospital admission (days)	Mild	0.102	0.864
	Severe	**0.049**	

Hughes	Mild	0.816	**0.032**
	Severe	**0.009**	

*Note:* Bold values indicate statistical significance.

The analysis of Table 2 demonstrates the correlation between the WHOQOL‐BREF questionnaire score and the ODSS‐LL score (*p* < 0.05), denoting how the loss of functionality, mainly related to ambulation, negatively impacts the patient’s perception of QoL.

Furthermore, an association was found between the time of hospital admission, the score on the Hughes prognostic scale at admission, and the score on the ODSS‐LL, showing that severe impairment of the LL from the onset, even if the search for hospital care is early, is associated with a worse functional prognosis (*p* < 0.05).

To evaluate the perception of QoL, qualitative and quantitative variables were correlated with the WHOQOL‐BREF questionnaire score.

Considering that this is a cross‐sectional study, to support the hypothesis that time is a significant factor in functional recovery, an ANOVA test analysis was carried out correlating the amount of time after GBS diagnosis with the score of ODSS and the WHOQOL‐BREF questionnaire. The conclusion was that the longer the time elapsed since the GBS, the better the scores for perceived QoL and functional capacity (Table 4).

**TABLE 4:** Correlation between WHOQOL‐BREF and quantitative factors.

	WHOQOL‐BREF	
	Corr (r)	*p* value
Age	−0.019	0.913
Time from onset of symptoms to hospital admission (days)	0.027	0.876
Time until the first dose of IG infusion	0.036	0.841
Hughes prognostic scale at admission	−0.191	0.264
Duration of hospitalization (days)	0.155	0.368
Time after the diagnosis of GBS	0.515	**0.020**

*Note:* Bold values indicate statistical significance.

The evaluation period post‐GBS diagnosis greater than 12 months (> 12 M) presents a positive correlation with the best score on the total ODSS and ODSS‐LL functionality scale. As for the perception of QoL, it was observed that the best score was obtained among patients who were evaluated between 6 and 12 months after the GBS condition. Regarding the timing of assessments, the observation that QoL peaks at 6–12 months while functional recovery continues past 12 months suggests a complex recovery trajectory. However, we must acknowledge a potential selection bias: patients with worse outcomes or persistent severe disability may be less optimistic after 12 months, which could reflect on the answers to the QoL questionnaire, and are less likely to attend late follow‐up.

To conclude the presentation of the results, we compared mortality rates across the distribution of qualitative factors using the chi‐square test. It should be noted that this assessment was based on a sample of 41 patients, given that—of the 46 patients who began the study—five did not attend follow‐up evaluations after hospital discharge.

As shown in Table 5, the Brighton criteria and the mEGOS score at admission are directly correlated with the risk of death. Among the five patients who died, 80% were classified as moderate/high risk at admission, whereas among the 36 survivors, 77% were classified as low risk.

**TABLE 5:** Comparison of deaths according to the distribution of qualitative factors (*N* total = 41).

		Survivor		Death		*p* value
		*N*	%	*N*	%	
Brighton criteria/mEGOS score admission	Low risk	28	77.8%	1	20%	**0.008**
	Mod/high risk	8	22.2%	4	80%	
Comorbidity	Yes	18	50.0%	4	80%	0.207
	No	18	50.0%	1	20%	
Gender	Feminine	14	38.9%	1	20%	0.411
	Masculine	22	61.1%	4	80%	
Neurological symptoms at admission	GMF ≥ 3	14	38.9%	1	20%	0.141
	GMF ≤ 3	7	19.4%	3	60%	
	Loss of strength restricted to the lower limbs	15	41.7%	1	20%	
Preceding symptoms	Dengue	2	5.6%	0	0%	0.482
	Diarrhea	14	38.9%	1	20%	
	Flu‐like symptoms	5	13.9%	2	40%	
	No symptoms	15	41.7%	2	40%	

*Note:* Bold values indicate statistical significance.

For a complete overview of the study, Table 6 demonstrates the baseline demographic, clinical, and treatment characteristics of all 41 patients (the 36 patients who were able to maintain regular outpatient medical follow‐up plus the 5 patients who died).

**TABLE 6:** Baseline demographic, clinical, and treatment characteristics of GBS patients.

Characteristics	Total (*N* = 41)	Completed the study (*N* = 36)	Death (*N* = 5)	*p* value
Demographics				
Age years (MEAN ± SD)	47.90	46.28 ± 16.19	59.60 ± 4,67	**0.0008**
GENDER (N. %)				0.411
Female	15 (36.6%)	14 (38.9%)	1 (20.0%)	
Male	26 (63.4%)	22 (61.1%)	4 (80.0%)	
Clinical features				
Preceding symptoms (N. %)				0.482
Dengue	2 (4.9%)	2 (5.6%)	0 (0.0%)	
Diarrhea	15 (36.6%)	14 (38.9%)	1 (20.0%)	
Flu‐like symptoms	7 (17.1%)	5 (13.9%)	2 (40.0%)	
No preceding symptoms	17 (41.5%)	15 (41.7%)	2 (40.0%)	
Duration of preceding symptoms, days (mean)	16.04	14.80	24.33	**0.036**
Hughes score at admission (mean ± SD)	2.88	2.75 ± 1.02	3.80 ± 1.10	**0.039**
mEGOS risk at admission (N. %)				**0.008**
Low risk	29 (70.7%)	28 (77.8%)	1 (20.0%)	
Moderate/high risk	12 (29.3%)	8 (22.2%)	4 (80.0%)	
Neurological symptoms at admission (N. %)				0.141
GMF ≥ 3	15 (36.6%)	14 (38.9%)	1 (20.0%)	
GMF < 3	10 (24.4%)	7 (19.4%)	3 (60.0%)	
Restricted to lower limbs	16 (39.0%)	15 (41.7%)	1 (20.0%)	
Comorbidities (N. %)				0.207
Present	22 (53.7%)	18 (50.0%)	4 (80.0%)	
Absent	19 (46.3%)	18 (50.0%)	1 (20.0%)	
Treatment and timing				
Time to hospital admission, days (mean)	7.29	7.08	8.80	0.444
Time to first immunoglobulin dose, days (mean)	9.92	9.65	12.25	0.299

*Note:* Bold values indicate statistical significance.

## 4. Discussion

Of the 36 patients who answered the WHOQOL‐BREF questionnaire, 15 of them (41.7%) had a score greater than 100, indicating they were very satisfied with their QoL. Eighteen patients had a score between 100 and 70, indicating they were moderately satisfied, and 3 had a score below 70, equivalent to being unsatisfied with their QoL. These data are similar to those described in the study carried out in Serbia by Berisavac and collaborators in 2020, although this study used a different QoL analysis scale [11].

Analyzing the statistical data, the heterogeneity of outcomes among patients with GBS is verified. The 8.7% mortality rate observed is higher than the 5% global average [12]. This discrepancy is likely due to the study’s sample size and the tertiary hospital setting, which handles more severe cases. An additional noteworthy finding of the study is that, when comparing the mean age of patients who died (59.60) with those who survived (46.28), a statistically significant association was observed between advanced age and mortality (*p* = 0.0008). This result suggests that older age may represent an important risk factor contributing to unfavorable clinical outcomes.

Another important observation is that greater neurological disability at hospital admission, as assessed by the Hughes scale, was associated with greater functional impairment in our cohort. This finding is consistent with previous studies showing that greater disability or weaker muscle strength during the acute phase of GBS is associated with poorer functional outcomes, as it was also shown in Rajabally’s publication [13], and the study by Demir and Köseoğlu, in which they specifically evaluated health‐related QoL and functional status in patients with severe GBS; however, they did not evaluate mortality [14].

In our cohort, a higher Hughes score at admission was associated with greater functional impairment on subsequent ODSS assessment, and a higher mEGOS was associated with the risk of death; although our study cannot establish an independent prognostic effect, this finding supports the use of admission neurological disability as a marker of disease severity. This is also shown in Habib’s research, in which the mEGOS score was considered a predictor of respiratory failure [15]. Considering this fact, it is important to report that a previous work by Fletcher and colleagues evaluated long‐term recovery and predictors of outcome in patients with GBS who required mechanical ventilation and did not interpret a Hughes score ≤ 3 as a predictor of a lower likelihood of requiring invasive mechanical ventilation; in fact, it was cited that the potentially wide ranges of functional disability within Grades 0 through 2 are difficult to distinguish and therefore to determine an association [16].

A negative correlation was observed between the score on the Hughes scale and the perception of QoL measured by the WHOQOL‐BREF scale, and although the analysis did not demonstrate statistical significance, this correlation contributes to the hypothesis that the higher the score on the Hughes scale at admission, the worse the prognosis after GBS, both from a functional point of view and from the perception of QoL.

Regarding preceding symptoms, diarrhea was not significantly associated with severity or outcome in our cohort. This finding should be interpreted cautiously because the sample was small. In established prognostic models for GBS, preceding diarrhea has been incorporated as a predictor of poor functional outcome together with age and muscle strength, rather than as a mortality predictor [8].

Evaluating the data between genders, there was no significant difference in severity or functional prognosis by sex in our cohort. Women had a higher median WHOQOL‐BREF score (103) than men (93); however, these values represent the findings of the present study and should not be attributed to the prognostic scoring study by van Koningsveld et al. [8].

The study by Khedr et al., carried out in Egypt, evaluated the perception of QoL after GBS and found that advanced age at the onset of the condition was decisive for a worse perception of QoL. In the present study, we found that 5 out of 10 patients over 60 years old were unsatisfied with their QoL, and one patient had an outcome of death [4].

In our cohort, a Hughes score > 3 at hospital admission was associated with mortality in univariable analysis. However, this finding should be interpreted cautiously because only five deaths occurred and multivariable adjustment was not feasible. Therefore, the present data do not establish the Hughes score as an independent predictor of mortality. The association is consistent with the broader literature showing that greater disability at admission is associated with poorer outcomes, but mortality‐specific inference should remain limited to this and Chio and collaborators’ study [12, 17‐18].

It is stipulated that the length of time period after GBS diagnosis is decisive for the improvement in functionality. Djordjevic et al. published a multicentric prospective study in 2020, in which it was demonstrated that there is a significant improvement in functional recovery throughout the first 6 months after GBS diagnosis [19]. In this study, the patients were divided into 3 groups: those evaluated with less than 6 months of the condition, those between 6 and 12 months, and those evaluated after 12 months of GBS diagnosis. Based on the results of the statistical analysis, we found that patients evaluated over 12 months had better scores on the ODSS functionality scale.

These data are also seen in the study by Forsberg et al.; in addition, it concludes that the evolution of improvement occurs mainly up to 24 months and that persistent disability at this time remains as a sequel 10 years after the syndrome [20]. The observed temporal discrepancy—where health‐related QoL peaks between 6 and 12 months while objective functional recovery (ODSS) continues to progress beyond 12 months—highlights a complex, nonlinear trajectory in GBS recovery, and it is also observed in a systematic review published by Darweesh and collaborators [21]. This early plateau in reported QoL may be largely driven by a psychological “response shift,” wherein patients adapt their internal standards, values, and coping mechanisms to accommodate residual physical deficits over time. Furthermore, this pattern may also be influenced by the fact that this is a cross‐sectional study, and by potential selection or attrition bias, as survivors with persistent, severe disability may experience psychological exhaustion and become less likely to attend late outpatient follow‐up evaluations.

Comorbidities have also been examined as potential predictors of in‐hospital mortality in larger administrative datasets [12]. In our cohort, 80% of patients who died had at least one comorbidity, although this association was not statistically significant (*p* = 0.207). Therefore, our data do not establish comorbidity as an independent predictor of mortality.

Factors such as the time from the onset of symptoms to the infusion of the first dose of immunoglobulin, and whether or not the patient had a well‐established episode of viral infection prior to the onset of GBS did not demonstrate a correlation with the outcome in the present study.

### 4.1. Strengths and Limitations to the Study

A notable statistical limitation of the present study is the inability to perform multivariable logistic regression modeling to establish whether clinical prognostic tools, such as the admission Hughes scale and mEGOS risk score, remain independent predictors of mortality after adjusting for potential confounders (such as age, preceding symptom duration, or baseline comorbidities). This analysis was strictly constrained by the low frequency of the primary outcome event, with five fatalities recorded among the 57 patients diagnosed with GBS (8.7% overall mortality rate). Standard statistical guidelines for regression analysis recommend a minimum threshold of 10 events per variable (EPV) to prevent model overfitting and unstable parameter estimates. Consequently, our prognostic assessments rely on univariable statistical evaluations (ANOVA and chi‐squared testing). Although both higher Hughes scale scores at admission and moderate‐to‐high mEGOS risk classifications demonstrated significant unadjusted associations with mortality in our cohort, which is consistent with the other larger studies cited in the article, a prospective investigation utilizing multicenter cohorts with higher event counts is required to perform adjusted multivariable testing and confirm their independent predictive capacity.

The absence of electrophysiological data also represents a limitation, as we were unable to perform electroneuromyography on the patients enrolled. Furthermore, the study did not differentiate between GBS subtypes, and electroneuromyography does not modify the treatment to be performed nor should alter the timing of treatment. The investigation focused on physical impairments, which were the most prominent and concerning complaints reported by patients.

Five patients were lost to follow‐up after hospital discharge, including one woman and four men, all of whom had been classified as low risk according to the Hughes prognostic scale and the mEGOS scale. These losses were voluntary, not associated with the primary outcomes, and the patients did not differ significantly from those retained in terms of baseline demographic or clinical characteristics, minimizing the likelihood of selection bias in a cross‐sectional study.

This study provides a unique contribution by presenting the patients’ perspective on outcomes and QoL, and by comparing these perceptions with objective measures of physical capacity through standardized scores and scales such as mEGOS and the Hughes scale. This comparison yields noteworthy findings and underscores the importance of considering patients’ perceived QoL, which should be regarded as a central goal in clinical practice.

In clinical practice, the Hughes scale and mEGOS may provide useful information for early risk stratification in patients with GBS. In our cohort, higher Hughes scores and moderate‐to‐high mEGOS risk were associated with mortality in univariable analyses. However, these findings do not demonstrate that the use of these scores or any specific management strategy based on them reduces mortality. Larger prospective studies are needed to determine their independent prognostic value and clinical utility.

## 5. Conclusion

This study underscores the significant impact of clinical characteristics at hospital admission on both functional recovery and QoL in patients diagnosed with GBS. The Hughes scale is a crucial contributor to analyze patient outcomes, with higher scores at admission correlating with more severe impairments in both domains.

One of the challenges in rehabilitation is time. The study data show that patients evaluated after 12 months of diagnosis had a better outcome compared to those evaluated before this period, highlighting the importance of extended follow‐up and the gradual nature of functional and psychosocial recovery in GBS.

Regarding mortality, a Hughes score > 3 at admission and moderate‐to‐high mEGOS risk were associated with death in univariable analyses. Given the small number of fatal events and the absence of multivariable adjustment, these findings should be interpreted as associations rather than independent prognostic effects. Early prognostic assessment may assist clinical risk stratification and counseling, but larger prospective studies are required to validate these associations.

Variables such as time until first dose of immunoglobulin infusion did not demonstrate statistical significance in this study.

Overall, these findings contribute to a deeper understanding of the clinical analysis of GBS outcomes and emphasize the need for personalized care strategies that consider the severity at admission and the timing of postdiagnosis evaluations. Further research, including larger cohort studies, is essential to refine prognostic models and improve long‐term management strategies for GBS patients.

## Author Contributions

Giuliana Pipolo: conceptualization; data curation; formal analysis; investigation; methodology; validation; visualization; project administration; and writing. Lara Santos Godoy: data curation; formal analysis; investigation; methodology; and writing. Eduardo Hummelgen: conceptualization; data curation; formal analysis; investigation; methodology; and writing. Carlos Arteaga Rodriguez: formal analysis; investigation; methodology; writing–review and editing. Otto Jesus Hernandez Fustes: conceptualization; data curation; investigation; methodology; formal analysis; project administration; supervision; and writing–original draft.

## Funding

No funding was received for this manuscript.

## Conflicts of Interest

The authors declare no conflicts of interest.

## Data Availability

The data that support the findings of this study are available from the corresponding author upon reasonable request.
